# Pretreatment elevated prognostic nutritional index predicts a favorable prognosis in patients with prostate cancer

**DOI:** 10.1186/s12885-020-06879-1

**Published:** 2020-04-29

**Authors:** Bin Li, Zheng Lu, Shengzheng Wang, Junqing Hou, Gang Xia, Heng Li, Bo Yin, Wei Lu

**Affiliations:** 1 Department of Urology, Xinyang Central Hospital, 1 Siyi Road, Shihe District, Xinyang, Henan 464000 People’s Republic of China; 2 Department of Urology, The First Affiliated Hospital of Zhengzhou University, Zhengzhou, Henan China; 3 Clinical Medical College of Henan University, Kaifeng, Henan China

**Keywords:** Prostate cancer, Prognostic nutritional index, Prognostic factor, Survival

## Abstract

**Background:**

The prognostic nutritional index (PNI), an immunity and nutrition based prognostic score, was correlated with clinical outcomes in different tumors. However, the prognostic significance of PNI has not been investigated in hormone sensitive prostate cancer (PCa). The objective of this study was to determine the prognostic significance of PNI in hormone sensitive PCa.

**Methods:**

Two hundred eighty PCa patients undergoing androgen deprivation therapy (ADT) as first line therapy at three centers were enrolled. The serum albumin levels and peripheral lymphocyte count were measured at the time of diagnosis. PNI was calculated as 10 * serum albumin (g/dL) + 0.005 * total lymphocyte count (per mm3). Patients were categorized in two groups using a cut-off point of 50.2 as calculated by the receiver-operating curve analysis. Univariate and multivariate cox regression analyses were performed to evaluate PNI as a favorable prognostic factor for progression-free survival (PFS), cancer-specific survival (CSS) and overall survival (OS). Prognostic accuracy was evaluated with the Harrell concordance index.

**Results:**

Multivariate analyses identified PNI as an independent prognostic indicator with respect to PFS (hazard ratio (HR) = 0.521, *p* = 0.001), CSS (HR = 0.421, *p* = 0.002) and OS (HR = 0.429, *p* = 0.001). Patients with elevated PNI had better clinical outcomes. The addition of PNI to the final models improved predictive accuracy (c-index: 0.758, 0.830 and 0.782) for PFS, CSS and OS compared with the clinicopathological base models (c-index: 0.736, 0.801 and 0.752), which included Gleason score and incidence of metastasis.

**Conclusions:**

Elevated pretreatment PNI was a favorable prognostic indicator for PCa patients treated with ADT.

## Background

Prostate cancer (PCa) is the most common malignance in men from United States [1]. Androgen deprivation therapy (ADT) is one of the initial treatments for patients with PCa [2].

In the present, the prognostic evaluation of PCa relies on the common indicators, such as PSA levels, TNM stage. Recent studies indicate that in addition to tumor cellular differentiation and biological behavior, the prognosis of cancer is also related to the immunological and nutritional status [3–6]. Jang WS et al. [7] reported that preoperative neutrophil to lymphocyte ratio was an independent prognostic marker for patients with PCa. Similarly, Langsenlehner T et al. [8] showed a significant association between platelet to lymphocyte ratio and prognosis of PCa patients who underwent radiation therapy. Serum albumin level, which is commonly used to assess the nutritional status, is an important prognostic factor in advanced cancer [9]. The specific relationship between nutrition and PCa still remained unclear [10], however, Kenfield SA et al. [11] reported that a healthy lifestyle might lower risk of lethal PCa, and Sejima T et al. [12] showed that low preoperative serum albumin was associated with lymph node metastases and biochemical recurrence of PCa in patients with radical prostatectomy, which indicated an important role of nutritional status in the prognosis of PCa.

The prognostic nutritional index (PNI), which is calculated on the basis of serum albumin levels and peripheral lymphocyte count, is a simple and comprehensive index to reflect the immunological and nutritional status. PNI was originally described by Onodera et al. [13] to evaluate the perioperative immunonutritional status and risk of post-surgical complications, and has been validated as an independent powerful prognostic factor for various types of cancer, including renal cell cancer [14], breast cancer [15], gastric cancer [16], colorectal cancer [17], hepatocellular carcinoma [18], pancreatic cancer [19], small cell and non-small cell lung cancer [20, 21], esophageal squamous cell cancer [22], and castration-resistant prostate cancer [23]. However, the value of PNI in the prognosis of hormone sensitive PCa has not been assessed.

Serum albumin levels and peripheral lymphocyte count are routinely performed before treatment for patients with PCa, and PNI is easy to be calculated. Accordingly, we investigated the prognostic significance of PNI in a cohort of hormone sensitive PCa patients from three Chinese centers.

## Methods

### Patient population

Three hundred twenty-two prostate cancer (PCa) patients underwent continuous ADT as first line therapy between Jan 2013 and Dec 2016 at three centers, including castration and antiandrogen therapy. Study exclusion criteria were inflammatory diseases, hepatopathy, autoimmune diseases, hematologic diseases, other types of cancer, cardiovascular and cerebrovascular diseases, and those patients lost to follow-up. Data from 280 patients were ultimately analyzed in this study. The study protocol was approved by the Ethics Committees from Xinyang Central Hospital, The First Affiliated Hospital of Zhengzhou University, and Clinical Medical College of Henan University, and all patients provided written informed consents.

Clinical and pathological characteristics were collected. All blood samples were measured before the prostate biopsy. PNI was calculated as 10 * serum albumin (g/dL) + 0.005 * total lymphocyte count (per mm3) [13]. The clinical metastasis was evaluated with radionuclide bone scan. PCa patients were divided into low-, intermediate-, and high-risk groups according to the EAU-ESTRO-SIOG guidelines [24].

Follow-up was assessed from the day of treatment to the day of death or last follow-up visit (Jun 2019). Overall survival (OS) was calculated from the day of treatment to the day of death or the last follow-up visit. Cancer specific survival (CSS) was defined as time between the day of treatment and date of death for PCa or the last follow-up visit. Progression was considered as castration resistance or death, and the castration resistance was evaluated according to the EAU-ESTRO-SIOG guideline [25].

### Statistical analysis

SPSS, version 19.0 was used to analyze the data. The receiver operating characteristic (ROC) curve analysis was performed to select the most appropriate cut-off point for PNI to separate the patients at high risk of cancer-related death. The correlation between PNI and clinical characteristics was assessed by the Wilcoxon signed rank tests for continuous variables, and the chi-squared tests for categorical variables. The Kaplan-Meier method was used for PFS, CSS and OS estimation, the log-rank test was taken to investigate the difference on survival. Stratified analyses were performed on the stratification of Gleason score or incidence of metastasis. The univariate and multivariate stepwise cox proportional hazard regression model was used to evaluate the effect of PNI on survival. The c-index was calculated to evaluate the predictive accuracy of model on basic clinical variables and for comparison after the addition of PNI using the R package “survival” [26]. A two tailed value of *P* < 0.05 was considered statistically significant.

## Results

Two hundred eighty patients were ultimately included in this study. Table 1 presented the baseline clinical characteristics of the patients. The median age of the patients was 76 years old (IQR, 67.25–79), and 131(46.79%) patients had bone metastasis at presentation. After a median follow-up of 46.0 months, 129 (46.07%) patients experienced disease progression, and 78 (27.86%) patients died, including 63 (22.50%) patients died of PCa.

**Table 1:** Clinical characteristics of prostate cancer patients treated with ADT (*n* = 280)

Parameters	No. of patients(%)
Age (median, interquartile range), years	76 (67.25–79)
PSA (median, interquartile range), μg/L	91.20 (29.26–189.00)
Gleason Score	
< 7	28 (10.00)
3 + 4	42 (15.00)
4 + 3	68 (24.29)
8	89 (31.78)
9	50 (17.86)
10	3 (1.07)
Metastasis	
No	149 (53.21)
Yes	131 (46.79)
Risk Stratification	
Low	1 (0.36)
Intermediate	26 (9.28)
High	253 (90.36)
PNI (median, interquartile range)	50.05 (46.46–53.55)
Progression-free survival	129 (46.07)
Cancer-specific survival	63 (22.50)
Overall survival	78 (27.86)
Types of ADT	
Orchiectomy + anti-androgen therapy	272 (97.14)
LHRN-a + anti-androgen therapy	8 (2.86)
Follow-up time (median, interquartile range), months	46.00 (31.00–59.00)

Abbreviations: *PSA* Prostate-specific antigen; *PNI* Prognostic nutritional index; *ADT* Androgen deprivation therapy; *LHRN-a* Luteinizing hormone releasing hormone-a

The best cut-off value of PNI for CSS as determined by ROC curve was 50.2. The patients in the low PNI group were significantly older than that in the elevated PNI group (*p* < 0.001). However, serum PSA level, Gleason score, risk stratification and incidence of metastasis were comparable in the two groups (*p* > 0.05). (Table 2).

**Table 2:** Clinical characteristics of prostate cancer patients according to PNI

Parameters	PNI		*P*-value
	< 50.2 (*n* = 146 52.14%) ≥50.2 (*n* = 134 47.86%)		
Age (median, interquartile range), years	77 (72–81)	75 (64–78)	< 0.001
PSA (median, interquartile range), μg/L	100.00 (35.5–216.00)	76.04 (26.60–135.61)	0.171
Gleason Score(≤7/> 7)	66/80	72/62	0.154
Metastasis (no/yes)	72/74	77/57	0.172
Risk Stratification (lowintermediate/high)	13/133	14/120	0.662

Abbreviations: *PSA* Prostate-specific antigen; *PNI* Prognostic nutritional index

**Fig. 1:**
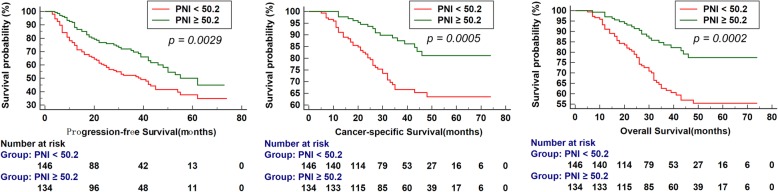
Kaplan-Meier curves for survival of prostate cancer patients according to PNI. a. Progression-free survival (PFS), b. Cancer-specific survival (CSS) and c. Overall survival (OS)

**Fig. 2:**
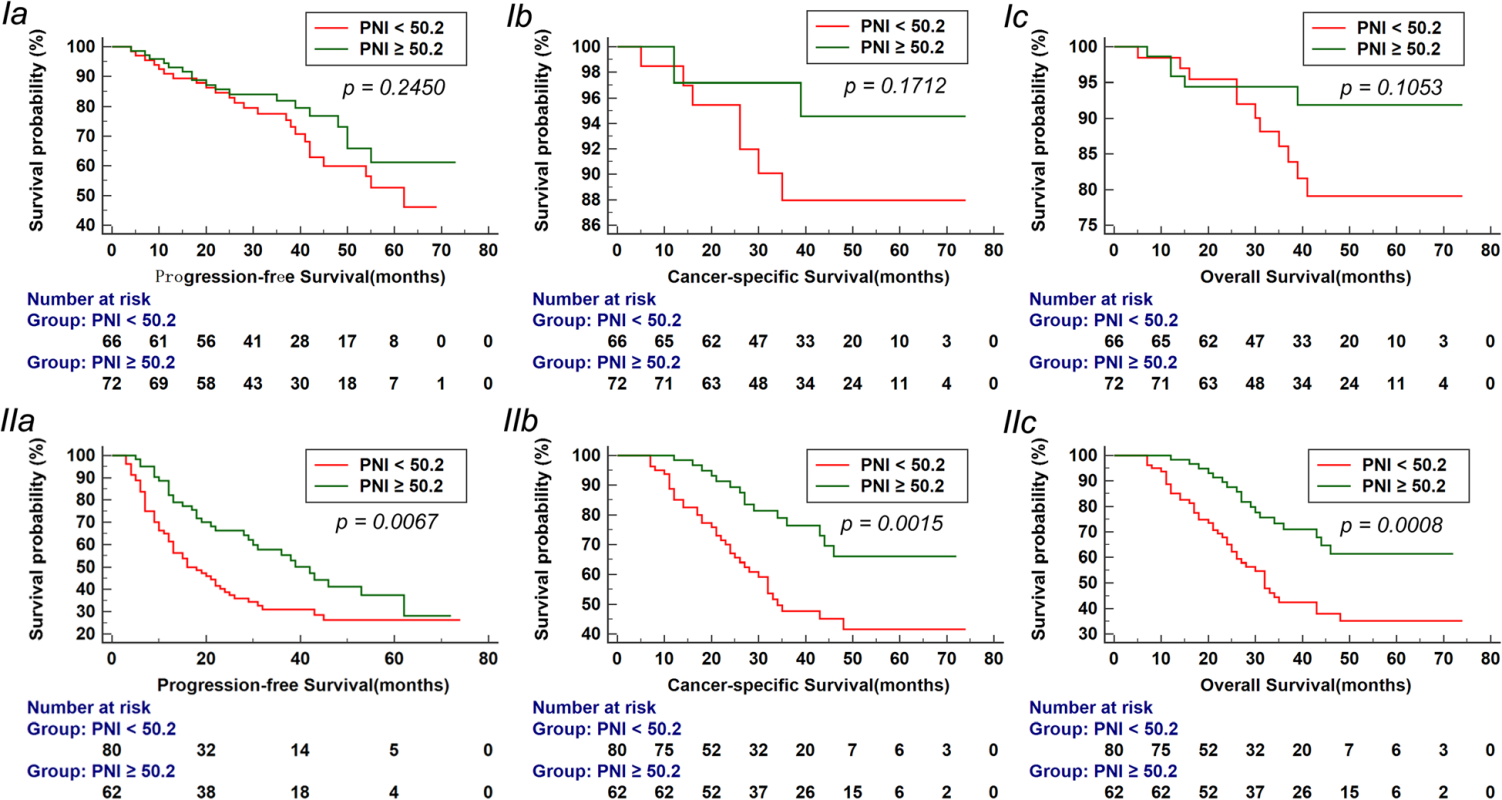
Kaplan-Meier survival curves stratified by PNI in prostate cancer patients with Gleason score ≤ 7(I) and Gleason score > 7(II). a. Progression-free survival (PFS), b. Cancer-specific survival (CSS) and c. Overall survival (OS)

**Fig. 3:**
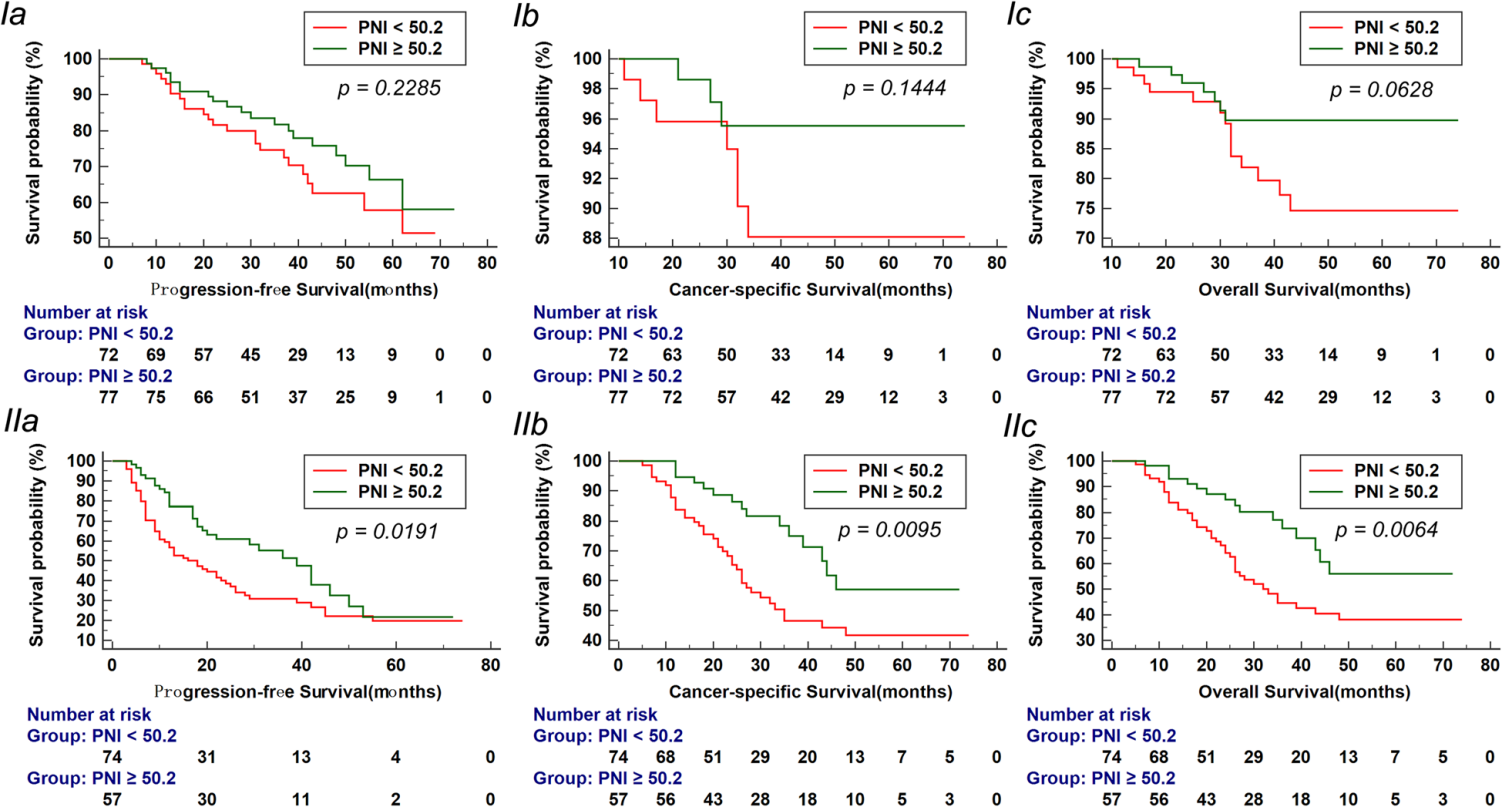
Kaplan-Meier survival curves stratified by PNI in prostate cancer patients with non-metastasis(I) and metastasis (II). a. Progression-free survival (PFS), b. Cancer-specific survival (CSS) and c. Overall survival (OS)

The Kaplan-Meier analysis showed significantly better PFS, CSS and OS in patients with elevated PNI than patients with decreased PNI (each *P* < 0.01, Fig. 1). As shown in Fig. 2 and Fig. 3, in the group of Gleason score > 7 or bone metastasis, the patients with elevated PNI had better PFS, CSS and OS (each *P* < 0.05). However, in the group of Gleason score ≤ 7 or non-metastasis, the prognosis was comparable in the two groups (each *P* > 0.05).

Univariate analyses demonstrated that elevated Gleason score, high incidence of metastasis, decreased PNI were associated with a worse PFS, CSS and OS (each *P* < 0.01), decreased age was a favorable predictor for PFS, not for CSS and OS (Table 3). Multivariate analysis indicated that age, Gleason score, incidence of metastasis and PNI were independent prognostic factors for PFS, while Gleason score, incidence of metastasis and PNI were independent prognostic factors for CSS and OS. The HRs of PNI were 0.521 (95% CI 0.355–0.765) for PFS, 0.421 (95% CI 0.243–0.728) for CSS and 0.429 (95% CI 0.263–0.698) for OS, respectively (Table 4).

**Table 3:** Univariate analyses of various clinical parameters in prostate cancer patients

Parameters	Progression-Free Survival		Cancer-Specific Survival		Overall Survival	
	HR (95% CI)	*P*-value	HR (95% CI) *P*-value		HR (95% CI)	*P*-value
Age (years)	0.969 (0.948–0.990)	0.004	0.971 (0.944–1.000)	0.050	0.988 (0.962–1.015)	0.396
PSA (μg/L)	1.000 (1.000–1.001)	0.228	1.000 (1.000–1.001)	0.273	1.000 (0.999–1.001)	0.611
Gleason Score		< 0.001		< 0.001		< 0.001
≤ 7	1		1		1	
> 7	2.907 (2.006–4.211)		6.425 (3.265–12.643)		4.740 (2.733–8.220)	
Metastasis		< 0.001		< 0.001		< 0.001
No	1		1		1	
Yes	3.528 (2.443–5.096)		7.920 (4.024–15.589)		4.122 (2.496–6.809)	
Risk Stratification		0.023		0.069		0.095
Lowintermediate	1		1		1	
High	2.428 (1.133–5.205)		24.120 (0.779–746.903)		2.357 (0.861–6.447)	
PNI		0.004		0.001		< 0.001
< 50.2	1		1		1	
≥ 50.2	0.590 (0.414–0.841)		0.391 (0.226–0.676)		0.407 (0.250–0.662)	

Abbreviations: *HR* Hazard ratio; *CI* Confidence interval; *PSA* Prostate-specific antigen; *PNI* Prognostic nutritional index

**Table 4:** Multivariate analyses of various clinical parameters in prostate cancer patients

Parameters	Progression-Free Survival		Cancer-Specific Survival		Overall Survival	
	HR (95% CI)	*P*-value	HR (95% CI)	*P*-value	HR (95% CI)	*P-*value
Age (years)	0.975 (0.953–0.997)	0.028		–		–
Gleason Score		< 0.001		< 0.001		< 0.001
≤ 7	1		1		1	
> 7	2.392 (1.638–3.495		5.073 (2.569–10.020)		4.003 (2.298–6.974)	
Metastasis		< 0.001		< 0.001		< 0.001
No	1		1		1	
Yes	2.930 (2.021–4.247)		6.091 (3.086–12.024)		3.245 (1.957–5.378)	
PNI		0.001		0.002		0.001
< 50.2	1		1		1	
≥ 50.2	0.521 (0.355–0.765)		0.421 (0.243–0.728)		0.429 (0.263–0.698)	

Abbreviations: *HR* Hazard ratio; *CI* Confidence interval; *PSA* Prostate-specific antigen; *PNI* Prognostic nutritional index

The c-index of the base model, including Gleason score and incidence of metastasis, for PFS, CSS and OS was 73.6% (IQR, 70.3–76.4%), 80.1% (IQR, 76.0–82.3%) and 75.2% (IQR, 71.4–78.1%). After the addition of PNI, the c-index for PFS, CSS and OS was 75.8% (IQR, 72.6–78.1%), 83.0% (IQR, 79.7–85.4%) and 78.2% (IQR, 74.4–81.7%), respectively which was higher than that of the base model (each *p* < 0.001).

## Discussion

The incidence of prostate cancer (PCa) in China is increasing in recent years [27], and the proportion of aged or advanced or metastatic PCa is higher than that in western countries, androgen deprivation therapy is the main treatment for these patients. Almost all the patients would experience castration resistance after ADT, to search for risk factors that could predict the prognosis of PCa patients receiving ADT is of great importance.

The serum albumin levels and peripheral lymphocyte count are routinely measured before the treatment of PCa. In this large cohort of PCa patients receiving ADT from three centers, we discovered that pretreatment elevated prognostic nutritional index (PNI) was a favorable prognostic indicator, independent of clinicopathological features and could significantly improve the predictive accuracy for prognosis, indicating it should be considered as an additional biomarker for PCa patients’ individual prognostic risk assessment.

The mechanism responsible for this observation remains unclear. The PNI represents a marker of immunity and nutrition. A decreased PNI reflects both malnutrition status and weak lymphocyte mediated antitumor immune response, which may both contribute to cancer progression and poor outcome. Malnutrition is highly prevalent in cancer patients, and may produce a great deal of negative consequences, such as impaired immune functions and quality of life, a higher degree of treatment-related toxicity and complications, reduced response to cancer treatment, lower activity level and shortened overall survival [28]. Sakurai K et al. [16] reported an association between low PNI with unfavorable outcomes for patients with gastric cancer at stages 1 and 2, and they speculated that malnutrition and immune compromise might promote proliferation and metastasis of peripheral blood circulating tumor cells (CTCs) in patients with early gastric cancer, increasing the risk of tumor recurrence. Therefore, malnutrition should be appropriately managed by structured collaboration between oncologists and clinical nutrition specialists [29]. Meta-analysis revealed that higher serum albumin level was associated with a better survival in patients with different tumors [30]. As part of systemic inflammatory response to the tumor or from the tumor itself, proinflammatory cytokines including interleukin-1, IL-6, and necrosis factor ɑ are released, which may modulate albumin synthesis by hepatocytes [31, 32], these cytokines are crucial for malignant transformation, neoangiogenesis and cancer progression [31], thus nutritional status could serve as a good indicator of prognosis for cancer. As to lymphocyte, it is one of the components of the immune system, which could inhibit tumor progression [33]. The HPV+ oropharyngeal cancer patients with pretreatment high circulating lymphocytes were reported with better prognosis [34]. Roth MD et al. [35] established a humanized prostate cancer model for understanding and manipulating tumor infiltrating lymphocytes, and found that the presence of tumor infiltrating lymphocyte was associated with a marked slowing of tumor growth although did not result in tumor eradication.

To date, it is controversial about the prognostic value of PNI in the clinical studies, some studies say yes [14–23], and others say no [36–38]. Sun K et al. [39] made a meta-analysis and showed that elevated PNI was a favorable predictor for OS, and the presence of post-operative complications, which indicated the association of decreased PNI with adverse clinical outcomes. The prognostic impact of PNI in many other cancers has been reported, on the contrary, its value in PCa is poorly investigated.

As far as I know, this is the first study to address the prognostic value of PNI in hormone sensitive PCa, and it revealed that elevated PNI was a favorable predictor for clinical outcomes. Moreover, we performed a stratified analysis and built a prognostic predictive model. In stratified analysis, we showed an association between decreased PNI and poor outcomes in patients with Gleason score > 7 or bone metastasis. In the patients with Gleason score ≤ 7 or non-metastasis, PNI could not predict the prognosis due to the small percentage of patients who reached the endpoints in this subgroup. The predictive accuracy for PFS, CSS and OS was significantly higher after the addition of PNI. In the meta-analysis by Sun K et al. [39], PNI was correlated with invasion depth and lymph node metastasis in gastric cancer, and TNM stage was the only clinicopathological feature associated with PNI in colorectal carcinoma. From the results of meta-analysis and aforementioned explanation, it seems that PNI should be associated with the clinicopathological features, however, the tumor features including serum PSA level, Gleason score, risk stratification and incidence of metastasis were similar in the two groups in our study, but similar to the report by Watanabe M et al. [40], the patients with low PNI had elevated age.

The same as all the retrospective studies, the first limitation of our study is its design. In an attempt to reduce the factors of influencing albumin levels and lymphocyte counts, we set up strict enrollment criteria, however, it is impossible to completely rule out other conditions that might cause immunological and nutritional changes in patients with PCa. Second, T and N stage were not showed for that 15% PCa patients with serum PSA level > 20 μg/L refused CT or MRI scan to evaluate tumor invasion depth and determine whether there was lymph node metastasis. Nonetheless, our data clearly indicated that PNI was an independent prognostic indicator for PFS, CSS and OS in PCa patients.

## Conclusions

Pretreatment PNI might be a novel prognostic predictor for PCa patients treated with ADT, the lower the PNI, the worse the prognosis. This biomarker should be considered in future prognostic risk assessment to improve the predictive accuracy.

## Supplementary information

**Additional file 1.**

## Data Availability

You can see the data and materials in additional file named “original data.xlsx”.
